# RNA World Modeling: A Comparison of Two Complementary Approaches

**DOI:** 10.3390/e24040536

**Published:** 2022-04-11

**Authors:** Jaroslaw Synak, Agnieszka Rybarczyk, Jacek Blazewicz

**Affiliations:** 1 Institute of Computing Science, Poznan University of Technology, 60-965 Poznan, Poland; jsynak@cs.put.poznan.pl; 2 European Center for Bioinformatics and Genomics, 60-965 Poznan, Poland; 3 Institute of Bioorganic Chemistry, Polish Academy of Sciences, 61-704 Poznan, Poland

**Keywords:** RNA world, partial differential equations, multi-agent systems

## Abstract

**Simple Summary:**

Despite years of dedicated research, scientists are still not sure what the first ”living” cell would have looked like. One of the most well-known hypotheses is the RNA world hypothesis, which assumes that, in the beginning, life relied on RNA molecules instead of DNA as information carriers and primitive enzymes. The population of such RNAs is made up of self-replicating molecules (replicases) that could make copies of themselves and parasite molecules that could only be copied by replicases. In this study, we further investigated the interplay between these hypothetical prebiotic RNA species, since it plays a crucial role in generating diversity and complexity in prebiotic molecular evolution. We compared two approaches that are commonly used to investigate such simple prebiotic systems, representing different modeling and observation scales—namely, microscopic and macroscopic. In both cases, we were able to obtain consistent results.

**Abstract:**

The origin of life remains one of the major scientific questions in modern biology. Among many hypotheses aiming to explain how life on Earth started, RNA world is probably the most extensively studied. It assumes that, in the very beginning, RNA molecules served as both enzymes and as genetic information carriers. However, even if this is true, there are many questions that still need to be answered—for example, whether the population of such molecules could achieve stability and retain genetic information for many generations, which is necessary in order for evolution to start. In this paper, we try to answer this question based on the parasite–replicase model (RP model), which divides RNA molecules into enzymes (RNA replicases) capable of catalyzing replication and parasites that do not possess replicase activity but can be replicated by RNA replicases. We describe the aforementioned system using partial differential equations and, based on the analysis of the simulation, surmise general rules governing its evolution. We also compare this approach with one where the RP system is modeled and implemented using a multi-agent modeling technique. We show that approaching the description and analysis of the RP system from different perspectives (microscopic represented by MAS and macroscopic depicted by PDE) provides consistent results. Therefore, applying MAS does not lead to erroneous results and allows us to study more complex situations where many cases are concerned, which would not be possible through the PDE model.

## 1. Introduction

Understanding how life might have begun is a fundamental problem in biology, requiring input from almost every discipline [1,2,3]. One of these is information theory, since evolution can also be perceived as a process whereby information flows from the environment into genetic material. Information is a key concept in evolutionary biology, and relates to quantifying the ability to make predictions about uncertain systems [4,5]. It must be physical, meaning that information cannot exist without a physical substrate to encode it [6].

The concept of information in molecular biology was developed based on Shannon’s theory [7,8]. Shannon [7] defined information in terms of signals, focusing on what it is while giving no explanation for how it comes into being. MacKay [9] went further and defined information in terms of what it does, linking it to meaning. Hence, genetic material (RNA or DNA) is not informational but, rather, through catalyzing processes or actions, promotes the propagation of organization. The information contained in RNA and DNA is structural, depends on the environment or context involved, and is not fixed in the same manner as Shannon selective information [5]. An explanation for where information comes from was provided by Kauffmann, who defined living organisms as autonomous agents able to act on their own behalf and to propagate their organization through autocatalysis [10].

Since much of the historic process of the origin of life has been lost and it is not possible to track back to our universal ancestor through the path of evolution, various theories on prebiotic evolution have been proposed. Among these, the RNA world hypothesis is probably the most extensively studied. It postulates an intermediate stage of life sustained by autocatalytic replication, where RNA played both genetic and enzymatic roles [11]. The discovery of catalytic RNAs and the fact that the ribosome itself is a ribozyme has added strong supporting evidence to this concept [12,13]. However, there is still an unresolved problem concerning the emergence of self-replicating RNA-only polymerase ribozyme (RNA replicase) from prebiotic conditions [14]. To complicate matters further, with the evolution of proteins, the catalytic properties of RNA were no longer needed and the ancestral replicases were entirely replaced by more efficient proteinaceous counterparts—namely, protein polymerase enzymes [15,16]. Therefore, in order to resolve the matter of whether the self-replication of RNA is possible, numerous studies have been dedicated to constructing and improving, through in vitro evolution, RNA polymerase ribozymes (RPR) that are able to conduct both de novo and templated non-enzymatic RNA polymerization [3,17,18]. Unfortunately, the RNA ribozymes that have been experimentally developed so far are longer than 100 nucleotides (the smallest one is 150 nucleotides long [18]), with a per-base error rate of a few percent; thus, they are unlikely to be discovered randomly [3,18,19,20,21]. Since it is not clear how such complex and large RNA replicases could have emerged from prebiotically produced short RNA molecules, many approaches to tackle this problem have been proposed [22,23]. One of the most plausible involves the spontaneous assembly of a functional RNA replicator from multiple phosphorimidazole-activated RNA oligomers (20–30 nt long) through iterative ligation, but the final yield of the full-length molecule is still rather low (0.5%) [18]. Nevertheless, despite the fact that none of the laboratory ribozymes are capable of replicating themselves yet, these experiments indicate that RNA enzymes operating in this way could have supported life in its earliest stages and might have triggered evolution.

Apart from the many serious obstacles involved, including but not limited to the supply of RNA building blocks and template replication in the absence of specialized enzymes, another challenge is the maintenance of genetic information in RNA sequences over many rounds of imperfect replication [24]. In order to survive, RNA polymerase must be copied faster than it is hydrolyzed and accurately enough to preserve its function and avoid the invasion of parasites (i.e., RNA molecules that do not possess polymerase activity but that can be replicated by RNA replicase) [25,26]. In the early stages of molecular evolution, due to the lack of reliable replication mechanisms, the mutation rate was likely very high and the critical amount of information could not have been stored in long RNA sequences; on the other hand, the short ones could not be efficient enzymes. Maynard Smith estimated that the maximum length of the RNA replicase is approximately 100 nucleotides, assuming nonenzymatic replication with a copying fidelity of one base of up to 0.99. In order to further increase this length, the copying fidelity would have to be increased, which requires the presence of specific enzymes [27]. This classic problem must have been faced by the prebiotic replicator systems. This is known as Eigen’s paradox and is often equivalently formulated as: no enzymes without a large genome and no large genome without enzymes [28,29,30,31]. As a solution to the problem mentioned above, Eigen proposed hypercycles, where overall genetic information is sustained as a set of diverse and different ribozymes, each carrying more reliably replicated and sufficiently smaller pieces of critical information [29,32]. However, Maynard Smith quickly raised an important objection to this theory, indicating that, without compartmentalization, there can be no selection that would give catalytic support to another molecule; thus, this property cannot be maintained [33]. Therefore, hypercycles are vulnerable to parasites, which will inevitably emerge through faulty replication and break down the cooperative loop. There seems to be a large class of such fatal mutant sequences, which implies that hypercycles are evolutionarily unstable [33,34].

The hypercycle theory was first formulated and studied in terms of ordinary differential equations (ODE) assuming the presence of a perfect mixed medium [24,28,29]. Later works showed that, in order to avoid a parasite-induced collapse, a spatial effect that limits interactions between molecules should be taken into consideration. This allows for multiple levels of selection, changes the dynamics of mutant invasion, and mitigates the problem of the system stability of cooperative replicators [34,35,36,37,38]. In [35], Boerlijst and Hogeweg propose a discrete two-level cellular automaton model (CA) to examine the stability and dynamics of diversity in space and time within simple prebiotic ecosystems and showed that the hypercycles in such systems may form traveling wave patterns [35,36,38,39]. Furthermore, Cronhjort and Blomberg [36] explored the same reaction–diffusion model, which was formulated in terms of partial differential equations (PDE), and reported that the section of parameter space affording resistance against parasites was smaller than in the CA model presented in [35].

Insights obtained from hypercycle analysis were generalized and extended by taking into account simpler replicator systems. Among the many models considered, a minimal replicator network consisting of one replicase and one parasite species (replicase–parasite system (*RP model* for short)) proposed by Hogeweg and Takeuchi [38,40] gained the most attention. In this system, contrary to hypercycles, replicases do not form any spatial patterns by themselves, but their interactions with parasites generate spiral waves. Moreover, traveling waves can generate new waves, and this continual process makes the RP system globally stable [38]. Hogeweg and Takeuchi modeled the dynamics of this system using simple differential equations, as well as within the framework of stochastic cellular automata (CA) with spatial extension [37,38]. The comparison of the dynamics of both models revealed different results [37].

The above-mentioned replicase–parasite surface model was further extended and verified in [41,42], where, in order to increase the model precision, a multi-agent-based approach (MAS) with more realistic assumptions regarding the movement of entities (diffusion) was applied. As in typical MAS implementation, it consisted of agents and their environment. The agent (replicase or parasite) was modeled as a set of attributes. In [41], each molecule was equipped with only two parameters, with *l* being the fraction of time spent in the folded state and *a* being the affinity towards replicases. Next, in [42], the RP model was modified by taking into account the RNA sequence (structure) information and a mutation rate close to the real one. The obtained results provided grounds for drawing the conclusion, which was that, in a prebiotic environment, RNA replicases could have appeared randomly, and that evolutionary selection can be observed even in a very simple network [42].

As discussed above, there are many modeling techniques that can be used to study prebiotic systems. In this paper, we focus on two modeling formalisms: multi-agent systems (MASs) and partial differential equations (PDEs). The PDE model represents a macroscopic description, where, instead of single molecules, in a well-mixed environment, their concentrations are considered. Here, three types of molecules are taken into account: replicases, parasites, and resources. These are the building blocks that are necessary for the population to grow. In contrast to the ODE model introduced by Hogeweg and Takeuchi [38], in our model, spatial dynamics (diffusion) and mutations associated with imperfect replication are taken into consideration. On the other hand, the MAS model represents a microscopic description, where a discrete population is composed of single particles (agents) interacting in a given dynamic environment. Here, in contrast to [41,42], a simplified model is considered, where each molecule is characterized by only one parameter connected with the affinity towards replicases.

Next, we conduct extensive simulation experiments for both models. Finally, we compare them in order to identify the general principles and rules governing their dynamics and evolution. We show that approaching the description and analysis of the RP system from different perspectives (microscopic and macroscopic) yields consistent results. It is worth noting that there are four levels of complexity described for the emergence of the first organic molecules [1]. This article focuses on stage δ, when polynucleotide chains capable of self-organization are formed.

## 2. Materials and Methods

### 2.1. RP Model

The replicase–parasite model (RP model) was initially developed by Takeuchi and Hogeweg [38] and later extended in [41,42], where it was simulated and analyzed using multi-agent systems (MASs). In this model, the population of RNA molecules consists of replicases and parasites. The former have the ability to catalyze the replication of other molecules, whereas the latter can be replicated but do not possess replicase activity. In what follows, replicases serve both as enzymes and replication templates, whereas parasites can only be used as templates.

In [42], every RNA molecule was characterized by two parameters: the fraction of time spent in the folded state and the affinity towards replicases. Here, in order to keep the mathematical formulation in terms of PDE as simple as possible, only one of these was considered. This parameter is denoted by *a* and refers to the affinity towards replicases. More specifically, it reflects the efficiency of the template.

### 2.2. Modeling with Partial Differential Equations (PDE)

The population of prebiotic RNA molecules was most probably dissolved in water [43]. Therefore, molecules (replicases, parasites, and resources) can be modeled using densities, which are the functions of both space coordinates and time. Replicases’ densities are denoted by *r*, parasites’ by *p*, and resources that are necessary for the population to grow by *n*. They are described with the following equations:

(1) r=r(x,y,z,t)

(2) p=p(x,y,z,t)

(3) n=n(x,y,z,t)

where *x*, *y*, and *z* are space coordinates and *t* denotes time. These variables are real numbers that are not bounded in theory. In practice, their values are limited to certain ranges, because every experiment/simulation has to take place in a finite area within a clearly defined time frame. The values of *r*, *p*, and *n* have to be non-negative, as they represent the densities.

#### 2.2.1. Replication

Replication is a third-order reaction. Three reagents (replicase, template, and a unit of resources) have to be present to trigger it. Its rate can be defined based on the law of mass action as follows:

(4) v=k[A][B][C]

where *k* is the reaction constant; [A], [B], and [C] are the densities of the reagents; and *v* denotes how many reactions occur per unit of volume and of time.

Since every successful replication produces a single copy of the template (one molecule), it can be interpreted as the change in their density per unit of time. In order to compute the aforementioned velocity, the reaction constant can be replaced with the template’s affinity towards replicases. Thus, in this model, the efficiency of the replication depends entirely on the template.

#### 2.2.2. Decay

It is assumed that every RNA molecule eventually decays and that this process is non-enzymatic. Therefore, the decay can be considered as a first-order reaction and its velocity can be computed as follows:

(5) v=k[A]

where *v* is the number of decay events per unit of volume and time, *k* is the decay constant, and [A] is the density of the molecule.

#### 2.2.3. Resource Formation

It is assumed that resources are created by the Sun, geothermal energy, or any other stable chemical process. Thus, the rate of resource formation is constant and denoted by $n_{0}$.

#### 2.2.4. Diffusion

The diffusion can be fully accounted for using Fick’s second law:

(6) $$\frac{d[A]}{dt}=D\nabla^{2}\left[A\right]$$

where [A] is the density of the molecule; *D* is the diffusion constant of the molecule *A*; and $\nabla^{2}$ is the Laplacian operator, which describes the differences in density in the immediate vicinity of every point.

#### 2.2.5. PDE Model and Its Assumptions

For the discussion of the PDE model presented in this paper, several parameters need to be defined. The resource diffusion constant is denoted by $D_{n}$, whereas the diffusion constants of all RNA molecules are assumed to be the same and are denoted by *D*. Parameter *a* stands for the affinity towards replicases. It is assumed to be constant for replicases ($a_{R}$) and parasites ($a_{P}$). Additionally, the replication is considered to be imperfect; thus, every replicase that is copied has a chance *m* to produce a parasite. Every RNA molecule in the analyzed system can decay, and its rate is denoted by $d_{R}$ for replicases and $d_{P}$ for parasites.

(7) $$\frac{\partial n}{\partial t}=n_{0}-nr\left(a_{R}r+a_{P}p\right)+D_{n}\nabla^{2}n$$

(8) $$\frac{\partial r}{\partial t}=\left(1-m\right)a_{R}nr^{2}+D\nabla^{2}r-d_{R}r$$

(9) $$\frac{\partial p}{\partial t}=ma_{R}nr^{2}+a_{P}nrp+D\nabla^{2}p-d_{P}p$$

Initial conditions can be given by the densities of replicases, parasites, and resources for every point in the simulation area at a given point in time $t_{0}$.

#### 2.2.6. The Solutions for a PDE Model

##### Well-Mixed Solution

First, it was checked whether such a system has an equilibrium, where all the densities are constant over time and space. In this case:

(10) $$\frac{\partial n}{\partial t}=0$$

(11) $$\frac{\partial r}{\partial t}=0$$

(12) $$\frac{\partial p}{\partial t}=0$$

(13) $$\nabla^{2}n=0$$

(14) $$\nabla^{2}r=0$$

(15) $$\nabla^{2}p=0$$

This leads to the following equations:

(16) $$0=n_{0}-nr\left(a_{R}r+a_{P}p\right)$$

(17) $$0=\left(1-m\right)a_{R}nr^{2}-d_{R}r$$

(18) $$0=ma_{R}nr^{2}+a_{P}nrp-d_{P}p$$

Equation (17) can be rewritten as:

(19) $$0=r[\left(1-m\right)a_{R}nr-d_{R}]$$

One of the solutions is for r=0, but there is no stable population of RNA molecules in this case (no molecules can be replicated). The alternative is:

(20) $$d_{R}=\left(1-m\right)a_{R}nr$$

In order to solve it in the case of *r*, two assumptions are necessary: m<1 and n>0. Both of these are obvious, because m=1 implies a completely ineffective replication of replicases, whereas, for n=0, there are no resources that would allow for any replication to occur.

(21) $$r=\frac{d_{R}}{\left(1-m\right)a_{R}n}$$

Taking into account Equation (18), this can be solved for *p*:

(22) $$p=\frac{ma_{R}nr^{2}}{d_{P}-a_{P}nr}$$

In the equation above, *r* in the denominator can be substituted (Equation (21)):

(23) $$p=\frac{ma_{R}nr^{2}}{d_{P}-\frac{a_{P}nd_{R}}{\left(1-m\right)a_{R}n}}$$

One *r* in the numerator can be substituted in the same manner:

(24) $$p=\frac{ma_{R}nrd_{R}}{\left(d_{P}-\frac{a_{P}nd_{R}}{\left(1-m\right)a_{R}n}\right)\left(1-m\right)a_{R}n}$$

This can be simplified to:

(25) $$p=\frac{ma_{R}d_{R}}{d_{P}\left(1-m\right)a_{R}-a_{P}d_{R}}r$$

Assuming that m>0, the numerator is positive; thus, the denominator has to be positive as well for the solution to be correct:

(26) $$d_{P}\left(1-m\right)a_{R}-a_{P}d_{R}>0$$

The inequality above can be written as:

(27) $$1-m>\frac{a_{P}d_{R}}{a_{R}d_{P}}$$

The last thing to prove is that there exists a correct value of *n*. Thus, we can denote:

(28) $$A=\frac{ma_{R}d_{R}}{d_{P}\left(1-m\right)a_{R}-a_{P}d_{R}}$$

If the inequality above is fulfilled, A>0. Thus, Equation (25) becomes:

(29) p=Ar

The only equation not taken into account yet is Equation (16). *p* can be substituted using the equation above:

(30) $$0=n_{0}-nr\left(a_{R}r+a_{P}Ar\right)$$

Or, in a shorter form:

(31) $$0=n_{0}-nr^{2}\left(a_{R}+a_{P}A\right)$$

Using Equation (21):

(32) $$0=n_{0}-n\frac{d_{R}^{2}}{{\left[\left(1-m\right)a_{R}n\right]}^{2}}\left(a_{R}+a_{P}A\right)$$

Solving the above for *n* results in:

(33) $$n=\frac{d_{R}^{2}}{{\left[\left(1-m\right)a_{R}\right]}^{2}n_{0}}\left(a_{R}+a_{P}A\right)$$

The solution is always positive; thus, if the inequality (27) is fulfilled (which implies that m<1), there is always a correct steady state.

**Special cases** There are two special cases that have to be analyzed separately. They will be described briefly.

1. m=0: replicases and parasites can coexist in any proportion, but only if $a_{R}d_{P}=a_{P}d_{R}$;
2. The denominator in Equation (22) is equal to 0. Unless the previous special case is also fulfilled, there is no solution.

##### Linear Stability

One of the most basic methods used to prove stability is linearization around the point of equilibrium; however, this approach is problematic when applied to the density of replicases (the system is still assumed to be well-mixed):

(34) $$\frac{\partial \frac{\partial r}{\partial t}}{\partial r}=2\left(1-m\right)a_{R}nr-d_{R}=\left[\left(1-m\right)a_{R}nr-d_{R}\right]+\left(1-m\right)a_{R}nr$$

If the density of replicases is not zero, Equation (20), which holds at the point of equilibrium, can be applied:

(35) $$\frac{\partial \frac{\partial r}{\partial t}}{\partial r}=\left(1-m\right)a_{R}nr$$

Excluding the cases where either m=1 or $a_{R}=0$ (replicases cannot replicate under such conditions), the right side of the equation is positive. This means that any change in the density of replicases will be further magnified by the system. Thus, a more advanced model is necessary to prove the equilibrium’s stability.

##### Evolution of a Well-Mixed Solution

The analysis of this case is necessary to prove the stability of the equilibrium found above. All densities are the same everywhere within the area, but they can evolve over time. Thus:

(36) $$\nabla^{2}n=0$$

(37) $$\nabla^{2}r=0$$

(38) $$\nabla^{2}p=0$$

Since the evolution of the system is deterministic and does not depend on the coordinates, it will remain well-mixed at all times.

Equations (7) and (8) take the forms:

(39) $$\frac{\partial n}{\partial t}=n_{0}-nr\left(a_{R}r+a_{P}p\right)$$

(40) $$\frac{\partial r}{\partial t}=\left(1-m\right)a_{R}nr^{2}-d_{R}r$$

Dividing every equation by $r^{2}$ and denoting $y=\frac{1}{r}$:

(41) $$\frac{\partial n}{\partial t}y^{2}=n_{0}y^{2}-na_{R}+na_{P}py$$

(42) $$-\frac{\partial y}{\partial t}=\left(1-m\right)a_{R}n-d_{R}y$$

Equation (42) can be solved for *n*:

(43) $$n=\frac{d_{R}y-\frac{\partial y}{\partial t}}{\left(1-m\right)a_{R}}$$

Taking the derivative over time:

(44) $$\frac{\partial n}{\partial t}=\frac{d_{R}\frac{\partial y}{\partial t}-\frac{\partial^{2}y}{\partial t^{2}}}{\left(1-m\right)a_{R}}$$

Equations (43) and (44) can be used to eliminate *n* and its derivative from Equation (41):

(45) $$\frac{d_{R}\frac{\partial y}{\partial t}-\frac{\partial^{2}y}{\partial t^{2}}}{\left(1-m\right)a_{R}}y^{2}=n_{0}y^{2}-\frac{d_{R}y-\frac{\partial y}{\partial t}}{\left(1-m\right)a_{R}}a_{R}-\frac{d_{R}y-\frac{\partial y}{\partial t}}{\left(1-m\right)a_{R}}a_{P}py$$

This can be written in the form:

(46) $$\frac{\partial^{2}y}{\partial t^{2}}=\left(d_{R}a_{P}p-n_{0}\left(1-m\right)a_{R}+\frac{d_{R}a_{R}}{y}\right)+\left(d_{R}-\frac{a_{P}p}{y}-\frac{a_{R}}{y^{2}}\right)\frac{\partial y}{\partial t}$$

In general (assuming that *p* is a parameter), the equation above takes the following form:

(47) $$\frac{\partial^{2}y}{\partial t^{2}}=P\left(y\right)+Q\left(y\right)\frac{\partial y}{\partial t}$$

This can be interpreted from the point of view of physics as an equation describing a pendulum. P(y) is the acceleration dependent on the position and Q(y) represents friction (acceleration acting in the opposite direction to velocity). There are some conditions that need to be fulfilled for this interpretation to be valid:

- P(y) has to have a root—an equilibrium point. Moreover, it has to be negative for any *y* bigger than it and positive for anything smaller;
- Q(y) cannot be positive.

Finding the root ($y_{E}$) of P(y) is trivial:

(48) $$d_{R}a_{P}p-n_{0}\left(1-m\right)a_{R}+\frac{d_{R}a_{R}}{y_{E}}=0$$

(49) $$y_{E}=\frac{d_{R}a_{R}}{n_{0}\left(1-m\right)a_{R}-d_{R}a_{P}p}$$

$y_{E}$ has to be greater than 0, since *y* as the inverse of replicase density cannot be negative. Thus:

(50) $$n_{0}\left(1-m\right)a_{R}-d_{R}a_{P}p>0$$

(51) $$p<\frac{n_{0}\left(1-m\right)a_{R}}{d_{R}a_{P}}$$

Finally, P(y) is a decreasing function for positive arguments; thus, it is negative for $y>y_{E}$ and positive for $y<y_{E}$.

The only thing left to check is the sign of Q(y):

(52) $$d_{R}-\frac{a_{P}p}{y}-\frac{a_{R}}{y^{2}}\leq 0$$

Multiplying by $y^{2}$:

(53) $$d_{R}y^{2}-a_{P}py-a_{R}\leq 0$$

This is a quadratic inequality that can be easily solved (*y* has to be positive):

(54) $$y\leq \frac{a_{P}p+\sqrt{a_{P}^{2}p^{2}+4a_{R}d_{R}}}{2d_{R}}$$

The biggest value of *y* fulfilling this inequality will be called “the critical point”.

(55) $$y_{C}=\frac{a_{P}p+\sqrt{a_{P}^{2}p^{2}+4a_{R}d_{R}}}{2d_{R}}$$

**Figure 1:**
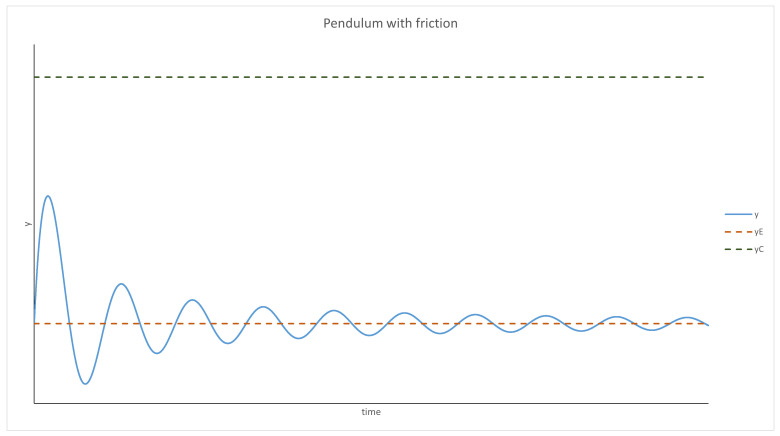
For *y* < *yC*, the system behaves like a pendulum with friction; it oscillates around the equilibrium, but these oscillations become weaker over time. Eventually, *y* becomes stable.

**Figure 2:**
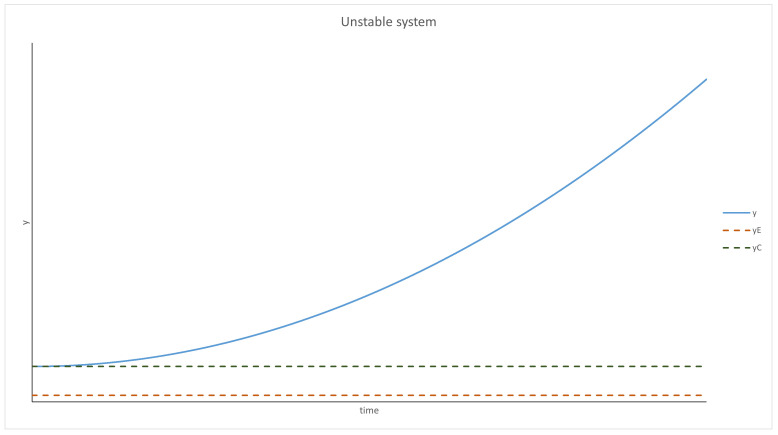
With *y* being past the critical point, the *Q* function becomes positive, thus increasing the velocity. *y* diverges to infinity, which means that the replicases die off (*y* is the inverse density of replicases).

Using the pendulum analogy, the behavior of the system can be predicted. For $y_{E}<y_{C}$ and $y<y_{C}$, the system behaves like a pendulum with friction; thus, it will oscillate around $y_{E}$ and will stop at this point eventually. The evolution of *y* over time is presented graphically in Figure 1 and Figure 2. It should be mentioned that *p* was treated as a parameter in this analysis (as mentioned at the beginning), and if the density of the parasites changes too quickly or by too much, this approximation will not be valid.

##### Mutation

If the inequality (27) is not fulfilled, the population of RNA molecules cannot be stable. Moreover, parasites do not have to keep their functions intact (like replicases), so they are expected to naturally evolve towards a higher $a_{P}$ over time. Thus, the inequality (27) cannot guarantee survival without any additional mechanisms. Previous models have shown that the system can still survive parasites’ mutations [38,41,42]. To explain this phenomenon, changes in the $a_{P}$ value have to be modeled explicitly; hence, the $a_{P}$ parameter has to become a function:

(56) $$a_{P}=a_{P}\left(x,y,z,t\right)$$

This function represents the average value of $a_{P}$ at any given point (and time). Obviously, another equation is necessary—one that can describe the evolution of the $a_{P}$ value over time. It can change due to three factors: diffusion, random mutations, and new parasites created as a result of faulty copied replicases. For ease of reference, it was assumed that the parasites derived from imperfect RNA replicase replication have the same $a_{P}$ as those already present; thus, this factor can be omitted. In order to form a proper equation, an important fact should be considered: $a_{P}$ cannot diffuse by itself. It is carried by parasites, so it cannot be designated directly by Fick’s second law. However, the value of $a_{P}p$ (the sum of $a_{p}$ values over all parasites) can be described as follows:

(57) $$\frac{\partial (a_{P}p)}{\partial t}=D_{P}\nabla^{2}\left(a_{P}p\right)+\frac{\sigma (x,y,z,t)}{\sqrt{p}}p$$

where σ is a random function that represents random changes in the $a_{P}$ value.

Due to the central limit theorem, for huge numbers of parasites molecules, σ should be a variable with a Gaussian distribution. It was assumed that the direction of a mutation is random, so, by default, $a_{P}$ neither increases nor decreases constantly. Since σ is a sum of many random variables ($a_{P}$s of all parasites), its changes should be inversely proportional to the square root of *p*. It corresponds to the empirical data—i.e., the larger the population, the slower it evolves. The equation above can be rewritten as:

(58) $$p\frac{\partial a_{P}}{\partial t}+a_{P}\frac{\partial p}{\partial t}=D_{P}\nabla^{2}a_{P}p+D_{P}a_{P}\nabla^{2}p+2D_{P}\vec{\nabla }a_{P}\cdot \vec{\nabla }p+\sigma \sqrt{p}$$

Since the decay and creation of new parasites does not affect the $a_{P}$ value, Fick’s pure second law for parasites can be used:

(59) $$\frac{\partial p}{\partial t}=D_{P}\nabla^{2}p$$

The Equation (58) becomes:

(60) $$p\frac{\partial a_{P}}{\partial t}+a_{P}D_{P}\nabla^{2}p=D_{P}\nabla^{2}a_{P}p+D_{P}a_{P}\nabla^{2}p+2D_{P}\vec{\nabla }a_{P}\cdot \vec{\nabla }p+\sigma \sqrt{p}$$

After solving it for $\frac{\partial a_{P}}{\partial t}$:

(61) $$\frac{\partial a_{P}}{\partial t}=D_{P}\nabla^{2}a_{P}+2\frac{D_{P}}{p}\vec{\nabla }a_{P}\cdot \vec{\nabla }p+\frac{\sigma }{\sqrt{p}}$$

##### Partial Differential Equations Model Robustness

All of the analyses above were carried out under certain assumptions that were necessary to draw any analytical conclusion. It is important to discuss what would change if the conditions were different. Several options are described below.

1. Diffusion: The presence of diffusion allows spatial interactions to occur. If there exists a stable, local equilibrium, this will be reached at every point and the system will become homogenous. However, for more complicated scenarios (such as the presence of mutation and the lack of a stable, local equilibrium), more complex structures can emerge and lead to the system’s survival. These cases are analyzed using computer simulations in the latter part of this article;
2. Complex formation: In this article, the replication of RNA molecules is treated as an instantaneous reaction, whereas, in reality, it takes time. In order to account for this, a new type of molecule can be added to the model—a complex of replicase with the template representing the ongoing replication—just as in [41]. However, the results obtained in the aforementioned work and this article show very similar behavior; hence, the addition of complexes does not seem to have much influence;
3. Different chemical reaction dynamics: reactions are assumed to follow the law of mass action. Due to the very complicated nature of complex molecules, especially in biology, replicases can behave differently depending on the density of resources and potential templates. The simplest case was assumed due to the fact that no functional replicase has been created in vitro as of yet; thus, their exact properties remain unknown. Changing the way the chemical reactions in the model work can radically change the outcome; for instance, replicases “programmed” to ignore parasites and only copy other replicases (but only if there are plenty of resources present) would be able to ensure the system’s survival without any additional mechanisms.

### 2.3. Description of the Partial Differential Equation Simulation Algorithm

In order to solve and analyze the results of the PDE, a simulation algorithm was implemented where infinitesimally small changes in coordinates (dx, dy, dz, and dt) were replaced with real numbers (Δx, Δy, Δz, and Δt). Thus, the equations were discretized. The state of the system was represented using a two-dimensional array. Every field represented a fragment of space and contained four variables:

- *p*: the density of parasites;
- *r*: the density of replicases;
- *a*: the average $a_{P}$ of parasites;
- *n*: the density of resources necessary for replication.

The whole algorithm is presented below in the form of the pseudocode (see Algorithm 1). It was implemented in C++ and parallelized using OpenMP. The global parameters of the simulation are listed in Table 1.
**Algorithm 1** Differential equation simulation  Initialize two 2D arrays—current and next  **for all** field *f* of current
**do**    Set f.p, f.r, f.k and f.n to 1.  **end for**  **for all** simulation step step
**do**    **for all** field *f* of current
**do**     $newreplicases=a_{R}*\Delta t*f.r*f.r*\left(1-m\right)*f.n$     
$newparasites=\Delta t*f.r*(a_{R}*f.r*m+f.a*f.p)*f.n$     
resourcesused=newreplicases+newparasites     **if**
resourcesused>0
**and**
resourcesused>n
**then**      newreplicases=newreplicases∗n/resourcesused      newparasites=newparasites∗n/resourcesused     **end if**     Compute average values for *f*’s adjacent neighbors: avg_r, avg_p, avg_n and avg_a.      Find the corresponing field of *f* in next - f2.     f2.r=f.r+newreplicases−d∗Δt∗f.r+D∗Δt∗(avg_r−f.r)     f2.p=f.p+newparasites−d∗Δt∗f.p+D∗Δt∗(avg_p−f.p)     new_a=f.a∗f.p+D∗Δt∗(avg_a−f.a∗f.p)     **if**
f.p>0
**then**      f2.a=new_a/f.p     **else**      f2.a=f.a     **end if**     $f2.n=f.n+n_{0}*\Delta t-newreplicases-newparasites+D_{n}*\Delta t*\left(avg\_n-f.n\right)$     Randomly change f2.a according to mutation probability distribution.     Set all negative variables of f2 to 0.    **end for**    Save results of the current simulation step.    Swap current and next.  **end for**

### 2.4. Description of the Multi-Agent Approach and Simulation Algorithm (MAS)

The PDE models the RNA population as a continuous solution, so, naturally, another approach was to model RNA molecules explicitly as agents. The first version of the simulation algorithm was described in detail in [41]. The algorithm presented below in the form of pseudocode (Algorithm 2) is based on the previous one.

#### 2.4.1. MAS Parameters

Several global parameters (Table 2) had to be used, along with a set of variables for a single agent. Every agent has the following parameters associated with it:

- Position;
- Type (replicase or parasite);
- $k_{P}$ (parasites only): the probability of being replicated.

#### 2.4.2. First-Order Reactions

The only first-order reaction in the simulation is the spontaneous decay of molecules. The probability of decay is the same regardless of the molecule’s age, and, for the decay rate *d*, can be computed as:

(62) $$p_{d}=1-e^{-d\Delta t}$$

In order to increase the efficiency, a different approach was chosen. In this approach, every new molecule is assigned a remaining life time (RLT) value, which is the number of simulation steps after which the molecule will decay. Statistically, this approach is equivalent to the previous one. RLT is computed using the following equation:

(63) $$RLT=\lfloor -\frac{\ln X}{d\Delta t}\rfloor +1$$

where *X* is a uniformly distributed random variable with values in the range (0;1].

#### 2.4.3. Second Order Reactions

These reactions can occur when two agents are close to each other and the circles representing them intersect. Depending on the types of agents, they have a certain probability of reacting:

- Two parasites: no reaction;
- Parasite and replicase: parasite becomes a template for replication and its $k_{P}$ becomes the probability of the reaction;
- Two replicases: the reaction occurs with the probability of $k_{R}$ (global parameter).

As a result, an exact copy of the template is created. If it is a parasite, then it is further subjected to mutation. It is important to note that $k_{P}$ and $k_{R}$ are not the same parameters as $a_{P}$ and $a_{R}$, because the former is the probability whereas the latter is the respective reaction rate.

#### 2.4.4. Mutation

Every new parasite is mutated with a probability of $m_{P}$. If a mutation occurs, then the parasite’s $k_{P}$ is changed by a value randomly selected from the uniformly distributed range $\left[-\frac{\delta }{2};\frac{\delta }{2}\right]$.

#### 2.4.5. Diffusion

The diffusion is simulated using Brownian dynamics (BD). At the beginning of every simulation step, every agent is moved by a random vector $\vec{\psi }$. This is calculated based on the following equation:

(64) $$\vec{\psi }=\sqrt{2D\Delta t}\vec{\xi }$$

where ξ is a random Gaussian vector with unit variance and mean equal to 0.
**Algorithm 2** Multi-agent algorithm  Initialize the agents’ positions randomly.  Initialize all parasites with equal $k_{P}$ values.  **while** (simulation time < time limit) **and** (there are both parasites and replicases present) **do**    Decrease the RLT time for each agent.    **for all** agents with the RLT = 0 **do**     Remove the agent.    **end for**    Randomize the order of all agents.    **for all** agent $x_{i}$
**do**     Initialize an empty set of neighbors *N*.     **for all** agent that overlaps $x_{i}$
**do**      Add it to the set of neighbors *N*     **end for**     **if**
$|N|>N_{max}$
**then**      Remove agent $x_{i}$ from the simulation.     **end if**     Randomize the order of *N*.     Move $x_{i}$ by a random vector (Gaussian distribution with variance 2DΔt).     **for all**
$n_{j}\in N$
**do**      **if** ($n_{j}$ or $x_{i}$ is a replicase) **and** reaction occurred (probability $k_{P}$/$k_{R}$) **then**      Create a copy of the template and place it in the same position.      Initialize new agents’ RLT value.      **if** New agent is a parasite **then**       Mutate new agent’s $k_{P}$.      **end if**     **end if**    **end for**   **end for**  **end while**

## 3. Results

The main goal of the conducted analysis and simulation was to test the most interesting case: where the inequality (27) is not fulfilled. Several scenarios were created to check possible parameter values that could lead to the system’s survival. In this regard, two modeling formalisms were compared—namely, multi-agent systems (Scenarios 1–8) and partial differential equations (Scenarios 9–10), which are described in both cases by corresponding and comparable sets of parameters in the case of the RP model. The results of the comparison were briefly compared and are discussed in a short summary at the end of the Results section.

### 3.1. Scenarios 1–8

The first eight scenarios were simulated using MAS, where the inequality (27) was not fulfilled. While the decay rate was the same for all molecules, the parasites had a greater replication efficiency. All parameters, except for *D* and $m_{P}$, were assigned default values (see Table 2). The goal of these simulations was to determine if the system could indeed survive and what the influence of the aforementioned parameters was on the final results of the simulations. The values of *D* and $m_{P}$ used for each scenario are presented in Table 3. Four values of diffusion were tested: slow (5), medium (10), fast (15), and very fast (10). There were also two possible values for mutation: slow (0.1) and fast (0.2).

**Figure 3:**
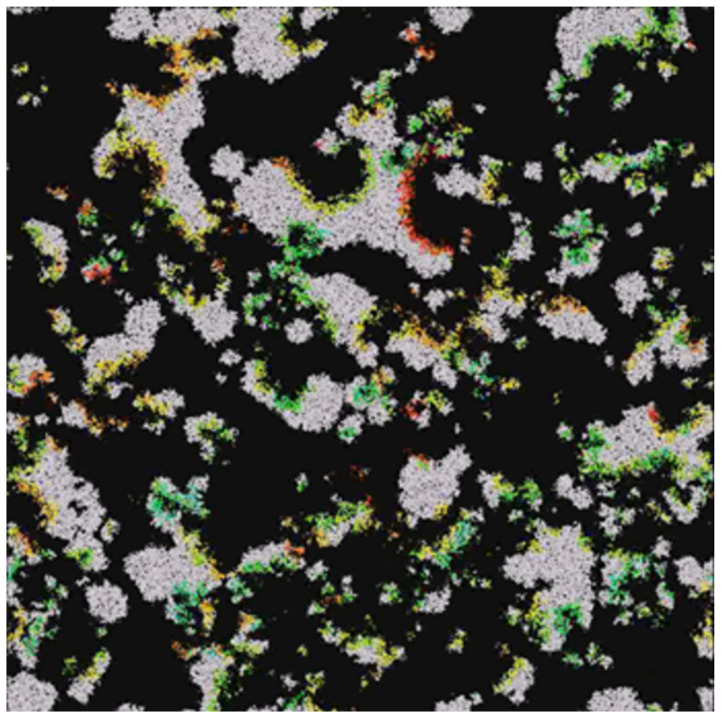
The waves formed in multi-agent systems. They represent various populations in different phases of the cycle. Replicases are gray, whereas parasites have colors denoting their reaction rate, with replicases ranging from red (very high) to yellow, green, blue, and purple (very low) [41].

**Figure 4:**
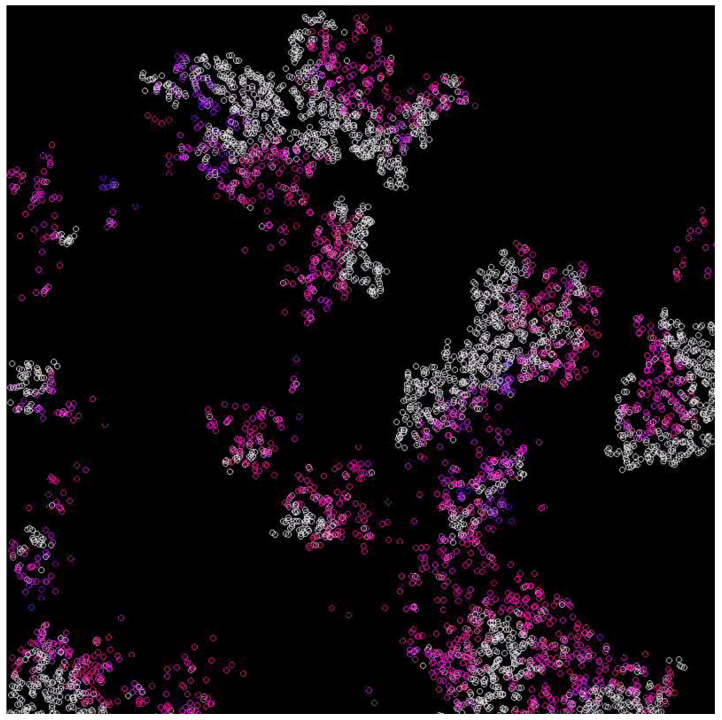
Waves for the diffusion constant equal to 5 and mutation rate equal to 0.1. Replicases are gray; parasites are purple.

**Figure 5:**
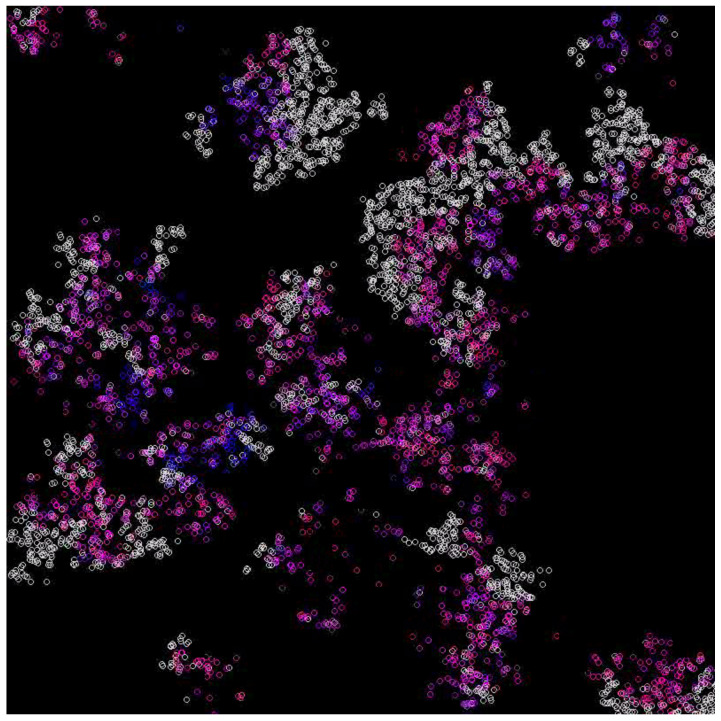
Waves for the diffusion constant equal to 5 and mutation rate equal to 0.2. Replicases are gray; parasites are purple.

**Figure 6:**
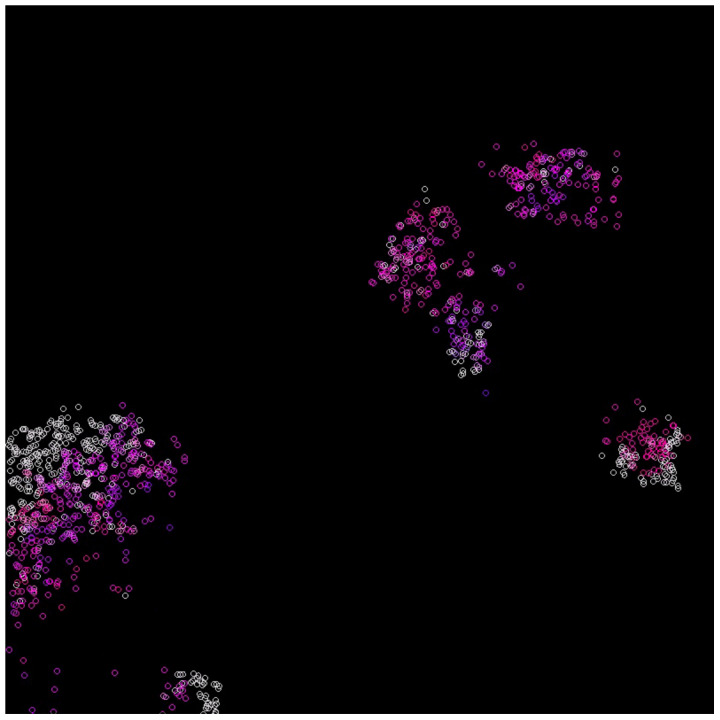
Waves for the diffusion constant equal to 10 and mutation rate equal to 0.1. Replicases are gray; parasites are purple.

In the case where the system survives, emerging waves can be observed (see Figure 3, Figure 4, Figure 5 and Figure 6). Replicases are colored in gray, whereas parasites are colored depending on their $k_{P}$ coefficient, ranging from red (0.0) to green (0.37) and blue (0.74) to magenta (1.0).

**Table 3:** Scenarios simulated using multi-agent system.

Diffusion Constant	Mutation Rate	Result
5	0.1	Alive (Figure 4)
5	0.2	Alive (Figure 5)
10	0.1	Alive (Figure 6)
10	0.2	Extinction
15	0.1	Extinction
15	0.2	Extinction
20	0.1	Extinction
20	0.2	Extinction

It can be observed that the parasites evolved locally towards a higher $k_{P}$ coefficient, but, at the same time, they always drove the population towards extinction. The whole system could survive because of the islands where, due to random chance, the parasites were too weak or there were not many of them. These islands can seed new populations, which evolve in exactly the same way as the initial one, creating a cycle. The most important variable that can be measured is the period of this cycle *T*, which depends on all other parameters. This function is most probably not analytical; thus, it has to be computed using simulations. If the velocity of the spatial expansion of the population is denoted by *v* and the smallest area that the population has to cover to be able to regrow after almost dying out is denoted by *P*, the condition for the system to form waves will be as follows:

(65) $$\pi {\left(vT\right)}^{2}\geq P$$

Deriving all three components mathematically is a subject for future work.

### 3.2. Scenario 9

**Figure 7:**
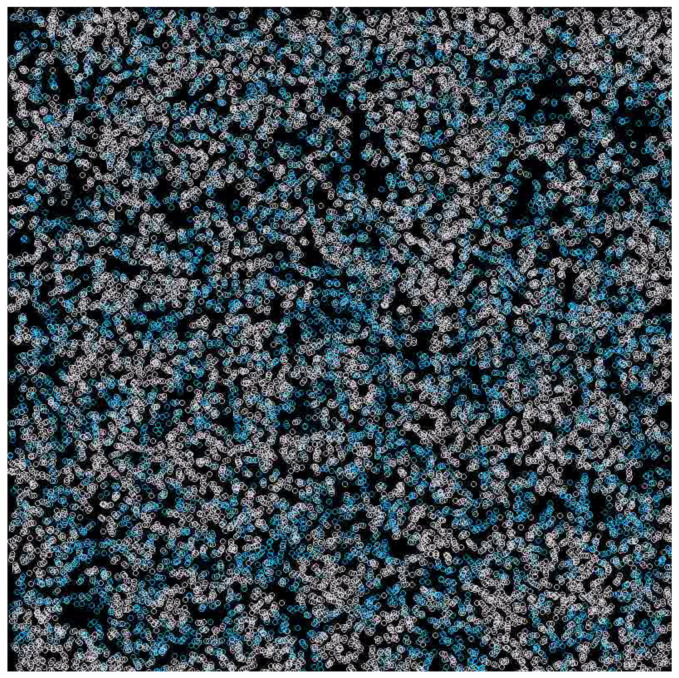
A well-mixed system, where replicases and parasites peacefully coexist. Replicases are gray; parasites are blue.

**Figure 8:**
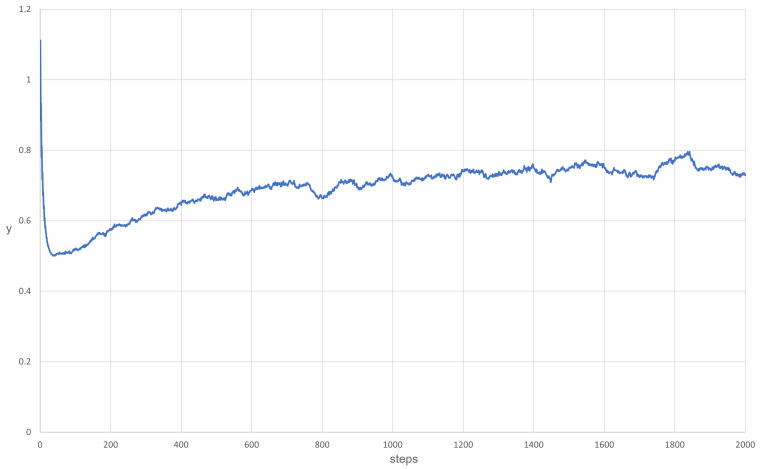
The evolution of y (the inverse of the number of replicases multiplied by 10,000 for readability) over time is very similar to the results obtained from Equation (46). The value of y begins to oscillate, but this oscillation is quickly slowed down.

The main goal of this scenario was to test a well-mixed system that is able to survive. The mutation was turned off, and the inequality (27) was fulfilled. The diffusion was set to 5.0, and the $k_{P}$ for all parasites was equal to 0.6. The results are shown in Figure 7. As a result, a coexistence could be observed. The system was able to survive and replicases and parasites were more or less mixed with each other (Figure 7). This simulation scenario further proves that RNA world can be well-mixed once the parasites are not strong enough to overtake it. Last but not least, the system behaved similarly to what Equation (46) predicted. This is illustrated in Figure 8.

### 3.3. Scenario 10

In this scenario, PDE was simulated. All global parameters were assigned default values and the simulation area was initialized, with all values being equal to 1.0.

**Figure 9:**
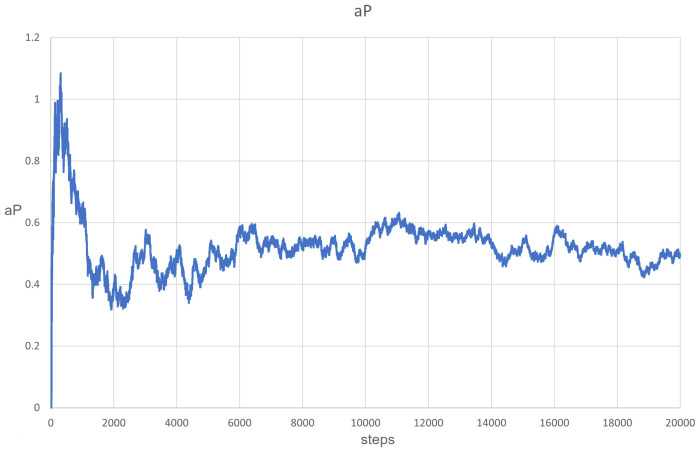
The evolution of aP at one particular point in space. This oscillated between values of 0.4 and 0.6.

**Figure 10:**
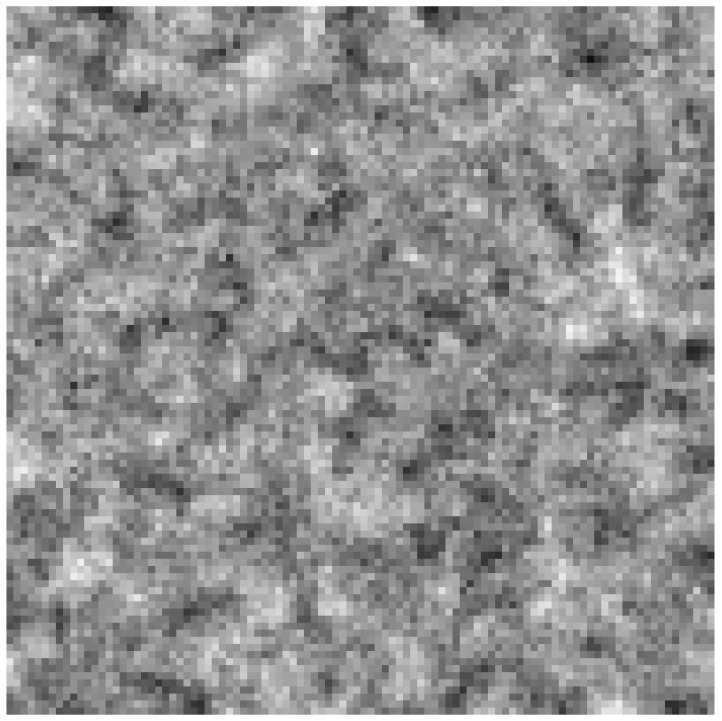
The waves formed in differential equation simulation, when inequality (27) is not fulfilled. Dark areas are devoid of replicases, whereas white areas have a high density of them.

The simulation of PDE clearly showed that $a_{P}$ (measured at one point in space) oscillated (Figure 9) between the values of 0.4 and 0.6, which could occur due to the changes in population composition and due to the cycles described above. It is also important that natural selection maintains the $a_{P}$ value in a safe range, despite more efficient parasites winning locally. Waves also form here, as can be seen in Figure 10.

### 3.4. Scenario 11

This scenario was almost identical to the previous one, but the parameter *m* was set to 0.1, thus rendering the replication far less reliable. The results were the same and waves formed, although the differences in the population density were actually greater.

### 3.5. Scenarios 12 and 13

**Figure 11:**
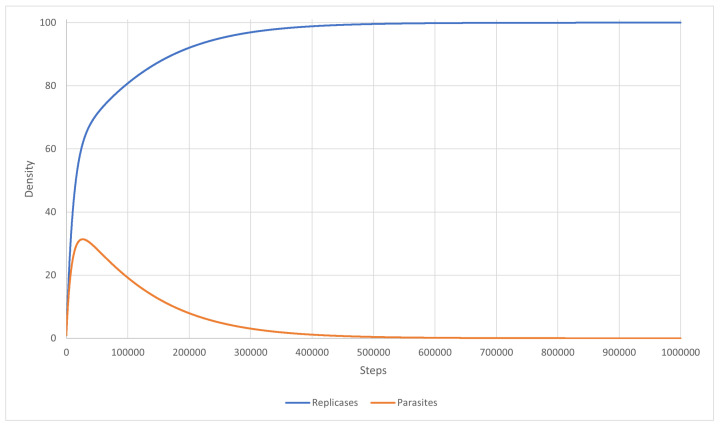
Scenario 12: the density of replicases and parasites. Replicases dominated the system because parasites had a lower reaction rate.

**Figure 12:**
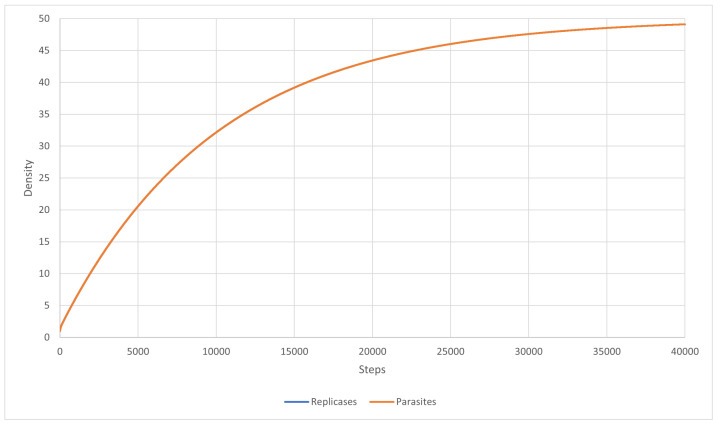
Scenario 13: the densities of replicases and parasites were equal, so the two graphs actually overlap.

Scenarios 12 and 13 correspond to Scenario 9. They were simulated to check if the inequality (27) could lead to system survival. The simulation was carried out using PDE with the mutation completely turned off. The system stabilized and became well-mixed, just as the equations predicted. In Scenario 12 (Figure 11), the reaction rate for parasites was equal to 0.9, which led to replicases dominating the entire system, whereas, in Scenario 13 (Figure 12), this reaction rate was 1.0 and both replicases and parasites reached equal densities.

### 3.6. The Summary of Scenarios 1–13

The analysis of Scenarios 1–13 clearly shows that, for both multi-agent simulations and partial differential equations, the system is locally unstable, as natural selection favors parasites with a higher affinity [41,42]. The population can expand if the parasites’ density is too low or if their $a_{P}$ is low enough that the inequality (27) is fulfilled. However, over time, the population of parasites grows and they become more efficient, eventually replacing the replicases and causing the death of the population. If the area covered by the population is large enough, in some places, $a_{P}$ should remain low and the population can start again from this spot. This causes the periodical behavior of the system, with it alternating between growth and decay. As many populations can exist near each other simultaneously and be in different stages of the cycle, the form of the waves in a multi-agent system looks like “population bursts”. This effect was observed by Takeuchi [38] and [41]. Simulations of the partial differential equations confirm this theory, as the same effect can be seen to occur.

## 4. Discussion

Simple prebiotic systems are usually investigated by dynamical models, which can basically be discrete or continuous in time and may also take spatial extension into account. Continuous time models are formalized as ordinary differential equations (ODE), whereas spatially structured ones can be treated in continuous space by partial differential equations (PDEs) or as cellular automata (CA) [25,38,44] in discrete space and time. More general approaches are based on multi-agent systems (MAS), which treat space similarly to CA but allow us to introduce more realistic diffusion based on Brownian dynamics [41,42]. The aforementioned models represent different modeling and observation scales—namely, microscopic (CA, MAS) and macroscopic (ODE, PDE). The microscopic view is more informative when considering localized dynamics, in which the interactions between single entities are relevant, whereas the macroscopic scale is appropriate when an insight into ensemble dynamics is required. Nevertheless, independently of the perspective taken, in general, models are expected to reproduce analogous phenomena, since they are deduced based on the similar assumptions. Hence, the goal of this research was to make a comparison between two representative modeling formalisms: multi-agent systems (MAS) and partial differential equations (PDE). These are described in both cases by corresponding and comparable sets of parameters in the case of a simple and well-studied replicator system (RP model).

It is worth mentioning that the efforts made towards understanding the synthesis of catalytically active RNA replicators and their population dynamics were thoroughly reviewed in [25]. Here, the authors checked several models of prebiotic systems against three main criteria: maintaining diversity, ecological stability, and evolutionary stability. One of the approaches analyzed was the metabolically coupled replicator system (MRCS), which assumes the presence of RNA replicators. The RP model can be considered as a special case of MRCS. Such systems could have been ecologically and evolutionary robust; however, it is still unknown whether short primitive RNA replicators could exist [25]. In [44], MRCS was further analyzed using a 2D toroidal lattice, which simulated a surface of a mineral deposit to which all RNA replicators were bound; hence, their mobility was limited. Here, RNA sequences and secondary structures were taken into account. Simulations showed that such systems can be ecologically and evolutionary stable, but are extremely sensitive to changes in their initial state [44].

Unfortunately, there is a wide gap between the discrete and continuous mathematical models proposed for the replicase–parasite system so far. The formulations existing in the literature are rather simple and focus on selected aspects of the RP model dynamics [34,38,45,46]. The first one, elaborated and later refined by Takeuchi and Hogeweg [34,38,45], considered the replicator system without the population structure or mutations. This was a simple ordinary differential equation (ODE) model that described a well-mixed system consisting of single replicase and parasite species. The study of its behavior revealed that the stable coexistence of parasites and replicases was possible, but, at the same time, clearly pointed out that a replicator system without a spatial population structure is evolutionarily unstable [38,45]. Another one, formulated in terms of ODE, focused on the emergence of genetic parasites within a self-replicating system and assumed a homogenous, well-mixed host–parasite system. The populations of replicases and parasites were environmentally limited by the same resources, whose capacity was introduced implicitly. Additionally, replicators were equipped with a defense mechanism against parasites that was capable of suppressing their replication. The analytical analysis of this system showed that, in order to reach stable equilibria, constructed models describing the coevolution of parasites and replicases cannot be too simple. Moreover, in order to stably coevolve, a parasite must possess a different reproduction strategy from a replicase. A parasite should depend on its host for both replication and building blocks [46].

Since no differential equation models that can coherently reflect the complexity of the replicase–parasite system exist in the literature, we introduced and thoroughly analyzed a comprehensive and sophisticated PDE model in which molecules—namely, replicases, parasites, and resources—are described as density functions. Additionally, the affinities of replicases and parasites towards replicases were assumed to be fixed and, in contrast to the abovementioned ODE models [34,38,45,46], diffusion, together with mutations as a consequence of imperfect replication, were taken into account. The population growth was limited through the environmental resources modeled as a function of both space coordinates and time. It should be mentioned that, as with every model, some simplifications and implicit assumptions have to be made. The reaction rates are treated as constants, which is an approximation used in the replicator equation employed to model interactions in prebiotic systems [47]. The densities in the model are assumed to be sufficiently low for molecules to diffuse freely. Last but not least, creating RNAs from a template is treated as an instantaneous reaction so as not to overcomplicate the model. Given all of the assumptions above, approximating the solution as continuous using PDEs should be accurate enough given the size of a molecule vs. the size of the simulated system.

First, an analysis of a well-mixed system assuming constant densities of all molecules was conducted and, as a result, the general inequality (27) that must be fulfilled for the system to reach a steady state was proposed. The stability of such a state was further analyzed using a pendulum analogy. Unfortunately, simulation-based analysis using MAS [41,42] has previously shown that the inequality (27) is generally not satisfied. Therefore, we extended our PDE model by taking into account more realistic assumptions through including mutations that must have been faced by prebiotic sets of molecules due to the lack of any error correction mechanisms. We presumed that only parasites can mutate as opposed to replicases, which should maintain their enzymatic activity. Thus, we modeled the evolution of the affinity of parasites toward replicases ($a_{P}$ parameter) as a function and investigated whether the system was able to survive. At first, we observed that, although many parasites went extinct, some islands in which $a_{P}$ had a low value occurred; thus, the inequality mentioned earlier (27) was fulfilled and the population was deemed to be internally stable and able to expand. However, over time, parasites were subjected to mutations and, as a result, their efficiency as templates ($a_{P}$ parameter) increased. Due to natural selection favoring parasites with high $a_{P}$ values, they began to out-compete replicases in being replicated, driving the population to extinction. In this situation, the abovementioned inequality (27) was no longer fulfilled. This observation is consistent with the results obtained using MAS simulations (see Scenarios 1–8 and [41,42]), where it was shown that parasites increased their average affinity towards replicases through evolution, thus proving that the selection favors better templates. MAS systems are considered a valid approach, since they are very flexible and can be used to model very complex molecular interactions. Their main drawback (which is shared by cellular automata) is their need to limit the number of molecules due to their finite computational power, since representing every molecule explicitly requires more resources. However, as simulations show, the scale can still be large enough for various effects to occur [41,44,45].

However, since mutations occur randomly, despite the fact that parasites that are better recognized by replicases are locally preferred, if the area covered by the population was large enough, during PDE simulation, there were some places that retained a low value of $a_{P}$. These small spots could begin to expand, and then the inequality mentioned above (27) was again fulfilled and the whole cycle started all over again. Given that many populations coexist nearby and very likely at different stages of the cycle, a characteristic oscillating behavior of the system, resembling waves, could be observed. A similar phenomenon, compared by the authors to traveling waves, was observed in CA simulations [34]. It is worth noting that the first experimental examples of evolving molecular, spontaneous RNA replication traveling waves were obtained and shown by [48].

In the MAS simulations of the RP system conducted in this work (see Scenarios 1–8), it could be noticed that parasites evolved locally towards having a higher probability of being replicated (denoted as $k_{P}$ coefficient), which led the population to extinction; however, the system as a whole could endure due to the small islands where the parasites were mild or in a small minority. However, the periodical behavior of the MAS system looked more like explosions of life because of the more continuous treatment of the space [41,42]. The observed spatial patterns consisted of populations of parasites and replicases represented by agents that were changing their location in a constant direction, giving an impression similar to that of waves in water. Such a wave was composed of replicases followed by parasites utilizing them and causing an empty space to emerge behind. The local extinction of parasites and the movement of new agents was responsible for the propagation of the wave [41]. Thus, it is clear that the MAS and PDE simulation results are consistent. It should be mentioned that the spatial aspect allowing for survival even with extremely efficient parasites is in agreement with previous results, where parasites could emerge due to a premature termination of replication, thus being shorter than functional replicases, which led to them undergoing replication more efficiently [49].

Furthermore, Scenarios 1–8 concerning MAS simulations were carried out mainly to investigate waves as a means of survival of the system. They focused on parameter range analysis in order to check their influence on the system collapse versus survival. The results of the simulations clearly show that the diffusion constant *D* is crucial regarding the system survival and evolution. If it is too high (see Scenarios 4–8), the propagation of the parasites with a high value of the $k_{P}$ coefficient is very effective and, eventually, the system will die as a result. Corresponding results were obtained through the PDE simulations shown in Scenarios 10–11.

Additionally, Scenario 9 was conducted to show that a stable coexistence of replicases and parasites is possible in a well-mixed system with no mutation modeled using MAS, where inequality (27) is fulfilled. The analogous simulations were performed for PDE (c.f. Scenarios 12–13). The results of these scenarios constitute further proof that an early prebiotic environment could be well mixed if the parasites are not strong enough to overtake it.

As has been mentioned above, simple self-replicating RNA molecules are very likely to have played a crucial role in the origin of life, and their co-evolution with parasitic RNAs produced through replication errors constituted the major driver in the life evolution. This concept is strongly supported by many theoretical models and computational simulation analyses, including the one presented in this paper. However, the experimental verification of the aforementioned models still remains a challenge, since it has not yet been possible to demonstrate the in vitro evolution of RNA from a realistic feedstock without exploiting any enzymes or environmental support [50,51,52]. To date, diverse molecular RNA polymerase ribozymes have been constructed and considerable efforts have been made to design interactions between them [18,19,20,23,52,53,54]. Many attempts have been made to elaborate and construct experimental RNA self-replicating systems that would allow us to investigate the host–parasite ecological dynamics [50,52,54,55]. In a recent study based on a system consisting of two classes of RNAs—namely, host and parasitic ones—and supported by the reconstituted translation system of *Escherichia coli*, where RNA was replicated by self-encoded RNA replicase (Qβ replicase), characteristic and regular population oscillation patterns due to the persistent replacement of dominant replicases and parasites were observed [52,55]. The detected population oscillation was consistent with the results shown in this study and in previous ones [38,41,42]. Notably, the parasitic RNAs that spontaneously emerged in the system as a result of recombination events and mutations introduced during replication into the internal replicase gene turned out to be descendants of a single RNA replicase, which is in compliance with our results reported in [42].

In the subsequent study using the aforementioned RNA replication system, a long-term replication experiment was performed. As a result, the further divergence of replicases and parasites into many RNA lineages was observed and a multiple replicator network was established [54]. Here, the irregularity of host and parasitic RNA concentrations was also detected, mainly in the early stage of evolution. In the later stage, it turned into a rather regular oscillation.

It is apparent that experimental systems are very important for testing whether theoretical predictions are relevant to the behavior of real evolving complex and cooperative networks of molecular replicators. Unfortunately, many outstanding challenges, such as efficiency, fidelity, and region specificity, remain to be overcome in order to produce a self-evolving and self-sustaining system in the laboratory that could provide insight into the dilemmas faced by life in the earliest periods of evolution on Earth and allow us to thoroughly verify the existing theoretical models and approaches.

## 5. Conclusions

In this paper, we proposed a new mathematical formulation for the replicase–parasite system in terms of partial differential equations (PDE). To the best of our knowledge, it is the first such comprehensive and sophisticated model that can coherently reflect the complexity of the RP system. We carefully analyzed the abovementioned PDE model both analytically and through extensive simulation experiments. Next, in order to identify the general principles and rules governing their dynamics and evolution, we thoroughly compared this model with a simplified version of the multi-agent system described by the corresponding set of parameters. We showed that approaching the description and analysis of the RP system using different modeling and observation scales (microscopic and macroscopic) allowed us to obtain consistent results. From this, it follows that, by applying a multi-agent approach, we can not only consider many complicated cases, such as the formation of protocells or self-assembled membranes, which would not be possible in the PDE model, but also avoid erroneous outcomes, since MAS and PDE lead to convergent results. Additionally, this clearly showed that mathematical modeling methods can be successfully applied for the description of RNA world dynamics and evolution.

The simulation results obtained for both models showed that natural selection favored better templates. Despite the fact that efficient parasites were winning locally, which meant that the system was locally unstable as natural selection favored parasites with a higher affinity, the system was able to survive due to some places where, because of random mutations, parasites were mild or in a small minority, and the population was able to restore itself. Therefore, in the simulations involving both models, we could observe spatial patterns resembling traveling waves, as seen in previous research [38,41].

As a continuation of the research reported in this paper, one may consider taking into account further mathematical and simulation analyses of the proposed PDE model, especially in the context of the period of the cycle, which can describe the oscillations of the system between growth and decay.

## Figures and Tables

**Table 1:** Global parameters used in the differential equation simulation.

Parameter Name	Default Value	Description
sizeX, sizeY	1000	Simulation area size
*d*	0.01	Decay rate
$k_{R}$	1.0	Probability of replicases reaction with replicases
Δt	0.1	Single step length (Δt in equations)
*D*	5.0	Diffusion constant
$D_{n}$	10.0	Resources diffusion constant
$n_{0}$	1.0	Resources production rate
*m*	0.0	Mutation of a replicase into a parasite probability

**Table 2:** Global parameters used in the MAS simulation.

Parameter Name	Default Value	Description
sizeX, sizeY	800	Simulation area size
agent_size	3.0	Radius of the circle representing an agent
$N_{max}$	4	Maximum number of neighbors
*d*	0.1	Decay rate
$a_{R}$	1.0	Affinity of replicases towards replicases
Δt	1.0	Single step length
*D*	15.0	Diffusion constant
δ	0.1	Parasite mutation speed
$m_{P}$	0.1	Parasite mutation probability

## Data Availability

Any data or material that support the findings of this study can be made available by the corresponding authors upon request.
